# Significant handling errors and education gaps regarding the use of the emergency kit among adult patients with Hymenoptera venom allergy 

**DOI:** 10.5414/ALX02476E

**Published:** 2025-01-22

**Authors:** Julia Zarnowski, Louise Wilkens, Regina Treudler

**Affiliations:** 1 Department of Dermatology, Venereology and Allergology, University Hospital Leipzig, Leipzig, and; 2 Institute of Allergology, Charité – Universitätsmedizin Berlin, Corporate Member of Freie Universität Berlin and Humboldt-Universität zu Berlin, Berlin, Germany

**Keywords:** insect venom allergy, hymenoptera, emergency kit, adrenaline autoinjector, compliance, handling errors, misinjections, anaphylaxis

## Abstract

Aim: In case of systemic anaphylactic reactions after Hymenoptera stings, patients should be provided with an adrenaline autoinjector (AAI). We aimed to evaluate the education and handling competence of patients in a real-world setting. Materials and methods: Patients with Hymenoptera venom allergy presenting for the first time in our clinic with a previously prescribed emergency kit including an AAI were interviewed using a standardized questionnaire and were asked to demonstrate the AAI use with a dummy. Results: 82 patients (62.2% female, mean age 52.0 ± 17.3 years) with allergy to wasp venom (85.3%), bee venom (9.8%), or hornet venom (4.9%) were included. 37.8% reported to have received a practical training on the AAI upon prescription. 59.8% of all patients showed significant handling errors which would have led to misinjections in 30.6%. Conclusion: Our data demonstrate a considerable lack of education, significant handling errors of the emergency kit, and a high risk of misinjections of the AAI. As the emergency kit is potentially lifesaving, the awareness for a sufficient education and training needs to be risen.

## Introduction 

Hymenoptera stings are the most common triggers of anaphylactic reactions in adults [1, 2, 3, 4]. Estimates assume that at least half of the general population will be stung by a wasp, bee, or hornet in their lifetime with most patients developing only local reactions while ~ 3% suffer from a potentially life-threatening Hymenoptera venom anaphylaxis [2, 4, 5]. In this regard, in Germany alone ~ 3.500 emergency medical interventions are required each year due to a Hymenoptera sting with 10 – 30 fatal outcomes per year [1, 2, 4]. In addition, it must be assumed that a high number of cases remains unreported. 

In case of a systemic reaction, physicians are advised by national and international guidelines to provide patients with an emergency kit containing antihistamines, corticosteroids, and – depending on the severity of the initial reaction and present risk factors – an adrenaline autoinjector (AAI) [1, 3]. Also, patients should receive written instructions and an emergency card on the components and handling of the emergency medication [1, 3]. 

As an anaphylactic reaction can be a serious, life-threatening event, which most commonly occurs outside of a clinical setting, an adequate education on the handling of the medication and compliance in carrying the emergency kit are crucial for preventing fatal events [3, 6]. 

Hymenoptera-allergic patients are commonly referred to our certified Comprehensive Allergy Center (CAC) for evaluation and induction of a venom immunotherapy, but we repeatedly observe a lack of education and improper use of the AAI in many patients. Thus, we aimed to evaluate the education and practical handling of the emergency kit in Hymenoptera venom-allergic patients in a real-world setting. 

## Materials and methods 

During the first presentation in our CAC, adult patients who had already been prescribed an emergency kit including an AAI were interviewed using a standardized questionnaire to assess the circumstances of the emergency kit prescription, in particular the duration, the components, and the information provided by the prescribing physician. All patients received a standardized clinical care including a medical history interview, oral consultation, and education on anaphylaxis and preventive measures. Before patients received a practical training, they were asked to explain and demonstrate the use of the AAI with a training pen without the intervention of the consulting physician in order to assess comprehension or application errors. The training device (Fastjekt (MEDA Pharma GmbH & Co. KG, Bad Homburg, Germany), Jext (ALK-Abelló S.A., Madrid, Spain), Emerade (Rechon Life Science AB, Limhamn, Sweden), or AnaPen (Owen Mumford Limited, Chipping Norton, United Kingdom and LYOFAL, Salon de Provence, France)) was chosen depending on the prescribed type of AAI or trained with various types in case of non-available information. 

Finally, there was a joint evaluation in which all detected errors were discussed, and remaining gaps in education or application could be closed. 

This observational study complies with ethical guidelines and was conducted in accordance with national law and with the Declaration of Helsinki of 1975 (in the current, revised version). Anonymous data evaluation was covered by informed consent (contract governing medical diagnosis/treatment and/or informed consent to anaphylaxis registry). Patient interviews and hands-on guidance were conducted as part of routine clinical care. 

Statistical analysis was performed with IBM SPSS Statistics Version 29.0. Descriptive results were shown as numbers and percentages. 

## Results 

### Patient characteristics 

82 patients (31 male; 51 female) with an average age of 52.0 ± 17.3 years and an average body mass index of 26.5 ± 5.2 kg/m^2^ (with 8 patients ≥ 100 kg) were interviewed. All suffered from Hymenoptera venom allergy (wasp: 85.3%; hornet: 4.9%; bee: 9.8%) with 1 (1.2%) patient having a large local reaction (Mueller grade 0), 10 (12.2%) Mueller grade I, 25 (30.5%) Mueller grade II reaction, 27 (32.9%) Mueller grade III, and 19 (23.2%) Mueller grade IV reaction. 

### Emergency kit (mis)prescription 

All patients (n = 82, 100%) had been prescribed with an AAI, most commonly Fastjekt (73.2%), followed by Jext (12.2%), Emerade and AnaPen (1.2%, respectively); 12.2% could not recall the prescribed type of AAI. 43.9% did not carry their emergency kit with them, of which over one third (36.1%) had a prior grade IV anaphylactic reaction and 5.6% suffered from systemic mastocytosis. 

68 (82.9%) had 1 AAI and 14 (17.1%) had 2 AAIs. 80.4% had a complete emergency kit consisting of an AAI, antihistamine (AH), and corticosteroid for oral intake while 9.8% had an AAI only, 6.1% an AAI with AH, and 3.7% an AAI with corticosteroids. 

With regard to the duration of supply with antiallergic medication, 29.3% of all patients stated that they possessed the emergency kit < 3 months, 39.0% 3 – 6 months, 9.8% 6 – 12 months, 4.9% at least 12 months, and 17.0% > 3 years. 

11 patients (13.4%) had already applied their AAI in the past with 2/11 even multiple times. 

According to the national guideline on the management of anaphylaxis, obesity or mastocytosis constitute an indication for the prescription of 2 AAIs, as higher dosages may be required for adequate cardiovascular response in these individuals [6, 7]. In our patient group, only 1/3 (33.3%) of patients with mastocytosis and 1/8 (12.5%) of patients with a body weight > 100 kg were equipped with 2 AAIs. A significant proportion of patients have been provided with a full emergency set, although this would not have been necessary according to the guidelines: 13.4% had received an AAI although they previously had only a large local reaction or a mild systemic reaction (grade 1 according to Ring and Messmer) *without* evident risk factors. On the other hand, although recommended by the national guideline on the diagnosis and treatment of Hymenoptera venom allergy, 19.6% of all patients did not possess a complete emergency kit containing an AAI, AH, and corticosteroid, and only 17.1% received the recommended written instruction as well as emergency card [3]. 

### Patient education and handling errors 

89.0% (73/82) of all patients reported to have received an oral instruction about the use of the emergency medication. Those who had received an oral instruction stated that the conversation lasted < 5 minutes (54.9%), while 25.6% had a consultation lasting 5 – 10 minutes, 11% for 10 – 15 minutes, and 2.4% > 15 minutes. 6.1% did not recall the duration of consultation. 

Only 17.1% received an emergency card as well as written instructions while almost half of all patients (48.8%) received neither. 19.5% received written instructions only, and 14.6% had an emergency card only. In total, 28.0% stated that they had additional questions or uncertainties after receiving their emergency kit prescription, which could not be addressed for time or organizational reasons. 

37.8% reported that the use of the AAI had been trained with them, while 62.2% had not received any practical demonstration. 

**Figure 1.**
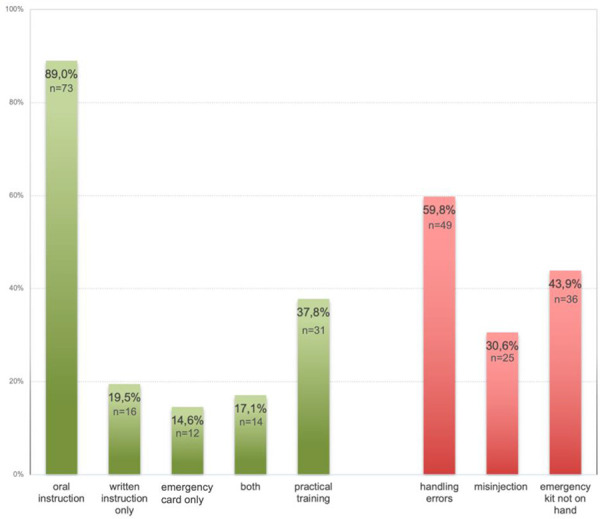
Percentages of successful patient education (green bars) and detected handling errors (red bars).

59.8% showed significant handling errors with 30.6% observed misinjections in the thumb and 24.5% failing to remove the protective cap, which would have resulted in the adrenaline not being injected (Figure 1). 

In our patient cohort, higher rates of handling errors were observed in patients who had neither received an oral or written information, nor a practical training and who did not carry their emergency kit with them. Also, older patients and patients with a higher severity grade of the initial anaphylactic reaction showed higher rates of application errors. The duration of emergency kit use or gender did not appear to influence the frequency of handling errors observed. 

### Discrepancies and misinformation 

Most patients had received the prescription by a dermatologist (36.6%), general practitioner (31.7%), or ENT specialist (22%). Less frequently, the emergency kit was prescribed by a pediatrician (3.7%), pulmonologist (2.4%), or a specialized allergy center (2.4%). One patient (1.2%) reported that “another specialty” prescribed the emergency kit. Most frequently, patients received an oral consultation on the use of the emergency kit from a dermatologist (45.2%), followed by a general practitioner (20.5%), allergist in a specialized clinic (17.8%), ENT specialist or pharmacist (6.9%, respectively), and pulmonologist or pediatrician (1.4%, respectively). 

A practical training was only received by 31/82 patients and was conducted most commonly in a specialized allergy center (54.8%), a dermatologist in private practice (9.4%), general practitioner or pharmacists (6.5%, respectively), pediatrician (9.7%), or ENT specialist (3.2%). 

**Figure 2.**
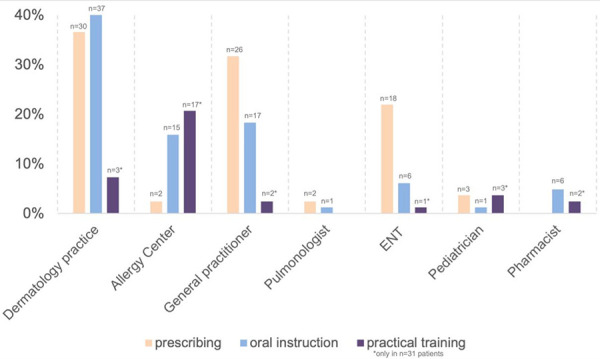
Discrepancies in prescription, oral instructions, or practical training between disciplines.

Altogether, a remarkable discrepancy was observed between different disciplines regarding the prescription rate, oral consultation, and practical training (Figure 2). 

As mentioned above, over one quarter of all patients was not able not discuss questions upon their initial appointment. As a result of the limited education and conversation, a total of 36.6% reported to have considerable uncertainties about when to take which medication. Additionally, several misconceptions have been reported or observed, such as an uncertainty about the amount of liquid (corticosteroid or antihistamines) to be taken; the lack of knowledge about the immediate application of AAI when dizziness or dyspnea occurs; the assumed impossibility to apply AAI through clothes; uncertainty about the AAI application site (inner thigh or deltoid reported by several patients) or an attempted subcutaneous AAI injection in the abdomen. 

## Discussion 

Our clinical survey revealed considerable deficiencies in the education and handling of the emergency medication in Hymenoptera venom-allergic patients, which would result in an inadequate application of the medication in the event of an emergency. We observed handling errors in 59.8% of all patients, which would have resulted in misinjections in 30.6% and to omitted injection in 24.5% due to failure in removing the safety cap. 43.9% did not carry the prescribed emergency kit with them. 

In a study by Özdemir et al. [8], 37% of patients did not carry the prescribed AAI with them and only 38% showed a correct application with the demonstration pen, whereby almost 52% failed to remove the safety cap. Similarly, Fischer et al. [9] showed that only 52% regularly carried their emergency kit with them with only 31% of patients being able to correctly demonstrate the use of their emergency kit in theory and practice. 50% of all patients who had been provided with an AAI misinjected the dummy in the thumb [9]. Other studies have also reported significant rates of incorrect administration of AAI pens with common mistakes being the failed removal of protective caps, not holding the AAI in place for several seconds or inappropriate choice of injections sites [8, 10, 11, 12]. As reported by Özdemir et al. [8], patients who did not carry the emergency kit with them were also less able to demonstrate how to use it and showed significantly higher handling errors. Other studies also emphasize the positive effects of (recurrent) practical trainings on the carry-on rate as well as correct handling of AAIs [8, 13, 14]. A study that evaluated the ability of correct AAI application of parents with food-anaphylactic children showed that 69% failed to correctly use the device. Interestingly, this study shows that a prior consultation with an allergist compared to a general practitioner was found to be significantly associated with a correct handling of the AAI [13]. As demonstrated by Brockow et al. [15] in a multicenter study, a structured anaphylaxis education program in adults and caregivers of anaphylactic children leads to a significant increase in knowledge and management skills compared to a control group. To our surprise, we encountered discrepancies between different physicians that prescribed emergency kits but failed to orally and/or practically consult and train the patients on the correct use of the emergency medication – patients were frequently trained in specialized allergy centers, although the primarily prescribing physicians were most frequently in a practice. An analysis of the German-based anaphylaxis registry showed a similar discrepancy in the prescription of indicated AAI devices, as 84% specialized allergy centers versus 37% of outside prescribers were following the European guideline recommendations [16]. Moreover, another study has concluded that although AAI prescription rates have been increasing and might reduce hospitalization rates in anaphylactic patients, many professionals themselves are not familiar with the application of emergency medication [17, 18, 19]. As summarized by Posner et al. [19], many U.S. paramedics as well as 35% of Australian pharmacists, 36% of surveyed pediatricians in Turkey, and 25% of medical personnel in the Toronto area were not able to correctly demonstrate AAI administration or were even at risk of misinjection [19, 20, 21, 22, 23]. 

We also observed a potential misinjection in the thumb when using the dummy in 30.6% of all patients. Although, as mentioned above, misinjection are also occurring in medical personnel due to a lack of training, and several evaluations show that rates of unintentional misinjections are rising in patients as well [19, 24, 25]. Although misinjections can potentially lead to an ischemic event at the misinjection site, most evaluations show a full recovery is most patients [19, 25, 26, 27]. The main problem in accidental misinjection, especially in most patient with 1 prescribed AAI, remains the lost adrenaline dose that is needed during an anaphylactic event [19, 25]. 

In our evaluation, 36.6% of patients reported to be insecure about the sequence and timing concerning the use of the emergency medication. Similarly, a study evaluating 78 patients receiving venom immunotherapy showed that 33.3% could not correctly answer all questions concerning their AAI (administration, prescription frequency, storage, transportation) [28]. Another study on wasp-allergic patients from Germany showed that up to 38% were unable to provide any information on the correct dosage of their emergency medication or would have misdosed [9]. 

Concludingly, a rapid intervention in case of a severe anaphylactic reaction in Hymenoptera venom-allergic patients is crucial to reduce morbidity and mortality. Therefore, patients need to be thoroughly informed about anaphylactic symptoms and the according management which should include education about preventive measures and trigger avoidance, as well as the use, storage, and carrying of the prescribed emergency medication [29]. Prescribing physicians need to ensure that patients are well informed and properly trained in correct administering emergency medication including the AAI, as recommended by national and international guidelines [1, 3, 6]. 

## Acknowledgment 

Clinician Scientist Programm, Universitätsmedizin Leipzig. 

## Authors’ contributions 

JZ was responsible for the concept of the study; all authors were equally involved in the acquisition, analysis and interpretation of data and to paper writing. All authors gave their final, pre-published approval of the version and agree to be accountable for all aspects of the work. 

## Funding 

This work did not receive any special financial support. 

## Conflict of interest 

RT received honoraria for lectures, congress fees or research support from ALKAbello, Viatris, Novartis, AbbVie, Pfizer, Sanofi-Genzyme, LeoPharma, Allmirall all outside the submitted work. JZ received travel grants from ALK Abello. LW received reimbursement of travel expenses from ALK Abello, outside the submitted work. In each case COIs are unrelated to the submitted work.
